# The Use of Acetazolamide to Prevent Acute Kidney Injury in Patients with Cancer on High-Dose Methotrexate Treatment: A Retrospective Pilot Analysis

**DOI:** 10.3390/clinpract14060205

**Published:** 2024-11-28

**Authors:** Lindon Lin, Tracey Batt, Gregory M. Peterson

**Affiliations:** 1 School of Pharmacy and Pharmacology, University of Tasmania, Private Bag 26, Hobart, TAS 7001, Australia; li.lin@alfred.org.au; 2 Pharmacy Department, Royal Hobart Hospital, 48 Liverpool St., Hobart, TAS 7000, Australia; 3 Haematology Department, Royal Hobart Hospital, 48 Liverpool St., Hobart, TAS 7000, Australia; tracey.batt@health.qld.gov.au

**Keywords:** high-dose methotrexate, cancer, acetazolamide, acute kidney injury, urinary alkalinization, risk factors, therapeutic drug monitoring

## Abstract

**Background:** High-dose methotrexate (HDMTX) chemotherapy is associated with a significant risk of acute kidney injury (AKI). Acetazolamide is thought to increase methotrexate solubility via urinary alkalinisation, potentially reducing the risk of crystalline nephropathy. A tertiary hospital has included acetazolamide in its HDMTX protocols, although data on the risks and benefits are limited. This study evaluated the role of acetazolamide in managing patients receiving HDMTX and identified risk factors for AKI. **Methods:** The retrospective cohort pilot study included consecutive hospital patients who received HDMTX (≥500 mg/m^2^). Data collected from digital medical records included demographics, comorbidities, methotrexate dosages and serum concentrations, and pathology results. The development of AKI was defined by the Kidney Disease: Improving Global Outcomes (KDIGO) criteria. Relationships between variables and AKI were initially assessed using Mann–Whitney U-tests and chi-square tests, and significant variables were further analysed using logistic regression to identify independent predictors of AKI. **Results:** Among 66 HDMTX treatment cycles in 31 patients, AKI occurred in 0/7 cycles with acetazolamide versus 14/59 cycles without (*p* = 0.33). Increasing age, the presence of hypertension, and concurrent use of beta-lactam antibiotics were associated with the development of AKI. Age was identified as the strongest independent risk factor for AKI (odds ratio 1.12, *p* = 0.034). **Conclusions:** Optimising management protocols, especially for older patients, is essential to reduce AKI risk during HDMTX therapy. While acetazolamide did not appear to reduce the risk of AKI, this pilot study was limited by a small sample size. Large randomised controlled trials are needed to assess efficacy and patient outcomes.

## 1. Introduction

Methotrexate is a widely used and cost-effective drug in cancer treatment, administered intravenously at doses ranging from 30 mg/m^2^ to 20,000 mg/m^2^ based on diagnosis [1]. High-dose methotrexate (HDMTX), typically exceeding 500 mg/m^2^, can lead to adverse effects such as nephrotoxicity and myelosuppression [2]. The incidence of acute kidney injury (AKI) in patients on HDMTX ranges from 2% to 12% [3]. AKI can cause methotrexate accumulation due to reduced clearance, leading to further toxicity and potentially fatal outcomes. 

Nephrotoxicity in HDMTX can occur through three main mechanisms. First, an allergic reaction to methotrexate can result in interstitial nephritis, and it is contraindicated if an existing allergy is known [4]. The second mechanism is direct toxicity to the renal tubules from methotrexate in the blood, which is dose-dependent [5]. The third mechanism is believed to be tubular obstruction caused by the precipitation of methotrexate and its metabolites. Since methotrexate is poorly soluble under acidic conditions, a urinary pH of less than 7 needs to be prevented to reduce the risk of nephrotoxicity [3]. Standard of care involves pre-hydration and urine alkalinisation with intravenous sodium bicarbonate.

Acetazolamide is a weak diuretic that blocks carbonic anhydrase in the renal proximal convoluted tubule, increasing bicarbonate excretion and urine pH [6]. Although not approved for prophylaxis against HDMTX-induced nephrotoxicity, acetazolamide may reduce the risk of crystal nephropathy. Increasing urinary pH reduces the risk of crystallisation and may enhance the renal clearance of methotrexate through reduced tubular reabsorption with the change in urine pH. The use of acetazolamide gained particular interest during times of shortages of the availability of intravenous sodium bicarbonate [7,8,9]. Some centres have developed and implemented outpatient HDMTX treatment protocols, including the routine use of acetazolamide, with significant potential benefits in terms of cost savings and improved patient experience [10,11].

In a retrospective study of 92 patients, Ku et al. showed a strong trend of preventing either AKI or delayed methotrexate elimination (>5 days), especially in males [6]. The Royal Hobart Hospital (RHH), the major public teaching hospital in Tasmania, Australia, has adopted the use of acetazolamide alongside HDMTX. Patients receive acetazolamide from the beginning of their HDMTX cycle until methotrexate serum levels have dropped sufficiently (to below about 0.15 µmol/L). This study retrospectively evaluated this off-label use of acetazolamide, aiming to identify the incidence and risk factors of AKI associated with HDMTX.

## 2. Materials and Methods

This was a single-centre retrospective cohort study. The analysis focused on consecutive patients undergoing HDMTX treatment (500 mg/m^2^ or higher). There were no exclusion criteria. Given the anticipated small sample size, this study was designed as a pilot study.

Details recorded included age, sex, height, daily weight, body surface area, diagnosis, comorbidities, medications and dosages, biochemistry data (e.g., serum creatinine (SeCr), estimated glomerular filtration rate (eGFR), electrolytes and methotrexate levels), and urinary pH. Baseline potassium, sodium, eGFR, and SeCr were recorded as the most recent levels available prior to HDMTX administration, typically from the morning of the treatment cycle. Baseline creatinine clearance (CrCl) was calculated using the Cockcroft–Gault equation [12], with ideal body weight replacing actual body weight for overweight patients. Post-HDMTX SeCr and peak weight were recorded as the highest values within 7 days post-HDMTX, while post-HDMTX CrCl, eGFR, serum potassium, and serum sodium levels were recorded as the lowest values within the same timeframe. Changes in these values were calculated by the difference between baseline values and the respective minimum or maximum values within 7 days post-HDMTX. During the administration of HDMTX, urinary pH was checked with each void or at least every 8 h. 

AKI was identified and graded based on the Kidney Disease: Improving Global Outcomes (KDIGO) criteria [13]. Delayed methotrexate elimination was defined as serum levels exceeding 15 µmol/L after 24 h, 1.5 µmol/L after 48 h, or 0.15 µmol/L after 72 h [14]. The presence of nephrotoxicity-increasing drugs in patients receiving HDMTX was defined as the administration of such drugs while methotrexate serum levels were above 0.15 µmol/L. These drugs include known nephrotoxic agents (e.g., angiotensin-converting enzyme (ACE) inhibitors/angiotensin II receptor blockers (ARBs), valaciclovir, and non-steroidal anti-inflammatory drugs), allopurinol, sulphonamides and penicillins, proton pump inhibitors, and ciprofloxacin.

Categorical variables were summarised using frequencies and percentages, while numerical variables were summarised as medians with a range or interquartile range (IQR). The Mann–Whitney U-test assessed the statistical significance of differences in numerical variables between cycles with and without acetazolamide prophylaxis, between cycles with and without AKI, and between cycles with and without delayed methotrexate elimination. Relationships between categorical variables were analysed using chi-square tests with Fisher’s exact *p*-values. Multiple logistic regression was used to test the independence of risk factors for AKI. Data management and analysis were conducted using Microsoft Excel and jamovi.

Ethics approval was granted by the Tasmania Health and Medical Human Research Ethics Committee (reference: 24419; 28 April 2021). 

## 3. Results

There were a total of 66 treatment cycles of HDMT: 28X were analysed, involving 31 individual patients (median number of cycles per patient = 2; range: 1–5), comprising 22 males (71%) and 9 females (29%). The median age of the participants was 64 years (IQR: 53–71 years). The median HDMTX dose per cycle was 3500 mg/m^2^ (IQR: 2695–3500 mg/m^2^), with a median administered absolute dose of 6200 mg (IQR: 5000 mg–7200 mg). Patient and cycle characteristics are summarised in Table 1. All patients received intravenous hydration, along with intravenous and/or oral sodium bicarbonate, aiming to maintain a urinary pH of at least 7. 

Acetazolamide (250 mg orally three times a day) was administered in seven of the studied cycles as an adjunct, with the intention of reducing the risk of AKI. Its use was decided by the clinical team. The acetazolamide group generally comprised older male patients (median age of 71 years vs. 61 years without acetazolamide; all were males). The two groups did not differ significantly with respect to kidney function (SeCr, CrCl, or eGFR) or other laboratory parameters at baseline or with respect to changes in kidney function, electrolytes, urinary pH, or weight gain following the administration of HDMTX. In the 7 days post-HDMTX, the median number of days with urinary pH dropping below 7 was 1 (IQR: 1–2) and 2 (IQR: 0–2) with and without acetazolamide, respectively (*p* = 0.86). The median increase in SeCr was 10.5 micromol/L (IQR: 3.5–18) and 8 micromol/L (IQR: −6–22) with and without acetazolamide, respectively (*p* = 0.41).

AKI was identified in 14 out of 66 cycles (21%). None of the cycles incorporating acetazolamide resulted in AKI incidents, whereas AKI occurred in 14 cycles (24%) without prophylactic acetazolamide (*p* = 0.33). The median age was higher in cycles with AKI (71 years, IQR: 65–72 years) compared to cycles without AKI (61 years, IQR: 52–70 years; *p* < 0.01) (Table 2). The median baseline estimated CrCl was slightly lower in cycles with AKI (85 mL/min, IQR: 61–99 mL/min) versus those without AKI (94 mL/min; IQR: 79–115 mL/min), although not statistically significant (*p* = 0.10). Among patients receiving beta-lactams, 67% developed AKI compared to 17% without beta-lactams (*p* = 0.02). 

**Figure 1:**
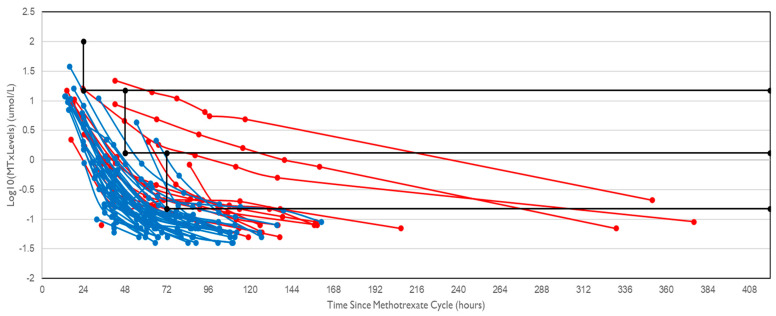
Comparison of methotrexate elimination between HDMTX cycles with (red) and without (blue) AKI. Data points within the black rectangular area indicate delayed methotrexate elimination, defined as serum levels above 15 µmol/L after 24 h, above 1.5 µmol/L after 48 h, or 0.15 µmol/L after 72 h.

AKI was associated with delayed methotrexate elimination; 67% of cycles with AKI had delayed elimination compared to 9% of cycles without AKI (*p* < 0.001). The serum methotrexate levels were plotted (Figure 1), and it was shown that the gradient of the plots was typically not as steep (i.e., elimination was slowed) in cycles associated with AKI compared to cycles where AKI did not occur. 

Binomial logistic regression identified age as a statistically significant independent risk factor for AKI (Table 3) with an odds ratio of 1.12 (12% increase in risk per year increase in age). The presence of beta-lactam antibiotics was marginally significant (*p* = 0.067).

Most patients with Grade 1 AKI recovered within 1–2 weeks, while those with Grade 2 and Grade 3 AKI took approximately a month to recover, with some experiencing ongoing renal impairment.

## 4. Discussion

In our pilot study, 21% of HDMTX cycles were associated with AKI, with 43% of these cases categorised as being Grade 2 or 3 (50% in each category). Increasing age was the key factor associated with AKI. This high incidence of AKI not only impacts chemotherapy and potentially cancer prognosis but also increases the risk of toxicities with other renally cleared medications and necessitates temporary or potentially permanent adjustments in managing other comorbidities. It is worth noting that we assumed that any cases of AKI were attributable to HDMTX given the temporal relationship with the administration of the well-known nephrotoxic agent. 

Several factors may have contributed to the elevated AKI rate in our cohort. Notably, patients with lymphoma, such as our sample, seem more susceptible to AKI compared to other cancers. A retrospective analysis revealed a 9.1% rate of renal toxicity in patients with lymphoma versus 1.5% in patients with osteosarcoma, despite significantly higher methotrexate doses used in sarcoma treatment [3,15]. However, lymphoma typically affects older patients (median age: 60–70 years), while osteosarcoma primarily affects younger individuals (<25 years) [16,17]. Given that our study identified increasing age as a risk factor for developing AKI, this would be a plausible explanation for the higher incidence of AKI in lymphoma patients compared to osteosarcoma patients.

Patients who received acetazolamide did not experience AKIs or delayed methotrexate elimination, unlike those without prophylaxis. Where acetazolamide was not given, AKI and delayed methotrexate elimination occurred in 24% and 25% of cycles, respectively. Our study lacked statistical significance due to sample size, so it does not definitively suggest a clear benefit of acetazolamide in reducing the risk of AKI. Unsurprisingly, AKI and delayed methotrexate elimination were often correlated. In cycles where AKI occurred, 67% had delayed methotrexate elimination. It is unclear from this study whether low urine pH led to delayed methotrexate elimination causing AKI or if crystalline nephropathy caused tubular obstruction leading to delayed methotrexate elimination. It is more likely that the nephrotoxic nature of methotrexate initially results in AKI, which can then lead to delayed methotrexate elimination, with further nephrotoxicity occurring due to methotrexate accumulation [18]. 

This study did not find baseline renal function to be a significant risk factor for AKI, likely due to the inclusion of patients without significant pre-existing renal insufficiency. Age emerged as the most significant predictor of AKI and delayed methotrexate elimination. Although renal function tends to decline with increasing age, both age and baseline renal function are known to be independent risk factors for AKI [19]. Weight gain was significantly greater in patients who developed AKI. It is recommended that patients’ fluid status should be regularly monitored when undergoing treatment with HDMTX. A weight gain exceeding 2 kg from admission may be an indication that HDMTX-induced renal toxicity is occurring [18,20].

This pilot study was an observational study with a small sample size. There could have been selection bias with respect to the clinical decision to administer acetazolamide. A recent systematic review and meta-analysis by Truong et al. included six observational studies and analysed four of these studies with the available data (total of 558 patients/cycles) [21]. It was concluded that there was no significant difference between acetazolamide and standard care treatment regarding urine alkalinisation time and the incidence of AKI in adult patients receiving HDMTX. 

## 5. Conclusions

An adequately powered randomised clinical trial is needed to evaluate the potential benefit of acetazolamide in protecting against HDMTX-induced AKI. Additionally, investigating whether acetazolamide increases the renal clearance of methotrexate, potentially reducing the clinical efficacy of treatment, is essential.

## Figures and Tables

**Table 1:** Summary of patient characteristics and HDMTX cycles.

Patient Characteristics	n (%)
**Sex (n = 31)**	
Males	22 (71%)
Females	9 (29%)
**Median age, years (IQR)**	64 (53–71)
Age > 70 years	11 (35%)
Age range	19–79 years
**Cancer diagnosis**	
Diffuse large B-cell lymphoma	20 (65%)
Central nervous system lymphoma	6 (19%)
Burkitt lymphoma	1 (3%)
Follicular lymphoma	1 (3%)
Grey zone lymphoma	1 (3%)
T-lymphoblastic lymphoma	1 (3%)
Primary mediastinal large B-cell lymphoma	1 (3%)
**Patient comorbidities**	
Hypertension (HTN)	13 (42%)
Type 2 diabetes mellitus (T2DM)	8 (26%)
Gastro-oesophageal reflux disease (GORD)	12 (39%)
**Cycles of HDMTX (n = 66 cycles)**	**n (%)**
**Presence of potentially nephrotoxic drugs**	
Furosemide	55 (83%)
Valaciclovir	52 (79%)
Allopurinol	11 (17%)
ACE-I/ARB	7 (10%)
Beta-lactam antibiotic *	6 (9%)
Ciprofloxacin	1 (2%)

* Amoxicillin, cefazolin, flucloxacillin, or piperacillin/tazobactam.

**Table 2:** Comparison of HDMTX cycles that were and were not associated with AKI.

Variables	Developed AKI	Did not Develop AKI	*p*-Value
N = 66	14 (21%)	52 (79%)	
Male	11 (23%)	37 (77%)	0.74
Median age, years (IQR)	71 (65–72)	61 (52–70)	0.01
Median dose, mg/m^2^ (IQR)	3500 (2927–3500)	3500 (2625–3500)	0.80
Type 2 diabetes mellitus	4 (20%)	16 (80%)	1.0
Hypertension	10 (38%)	16 (62%)	0.012
Acetazolamide	0 (0%)	7 (100%)	0.33
ACE inhibitor/ARB	2 (29%)	5 (71%)	0.63
Allopurinol	3 (27%)	8 (73%)	0.69
Beta-lactam	4 (67%)	2 (33%)	0.016
Ciprofloxacin	1 (100%)	0 (0%)	0.21
Furosemide	11 (20%)	3 (80%)	0.69
Valaciclovir	12 (23%)	40 (77%)	0.72
Delayed methotrexate elimination	10 (67%)	5 (33%)	<0.001
Median baseline SeCr, micromol/L (IQR)	71.5 (56.3–79)	64 (57–82)	0.84
Median post-HDMTX SeCr, micromol/L (IQR)	118 (112–184)	71.5 (59–86.5)	<0.001
Median baseline CrCl, mL/min (IQR)	85 (61–99)	94 (79–115)	0.10
Median post-HDMTX CrCl, mL/min (IQR)	37.5 (36–52)	91 (71–107)	<0.001
Median baseline eGFR, mL/min/1.73 m^2^ (IQR)	91 (82–99)	99 (90–108)	0.35
Median post-HDMTX eGFR, mL/min/1.73 m^2^ (IQR)	48 (34–62)	91 (81–100)	<0.001
Median days where urine pH < 7 (IQR)	2 (1.25–3)	2 (0–2)	0.06
Median gain in weight post-HDMTX, kg (IQR)	5.5 (3.13–8.22)	3.3 (2.2–4.4)	0.02

**Table 3:** Results for the logistic regression of risk factors associated with AKI.

Risk Factor	Odds Ratio (Adjusted)	95% Confidence Interval	*p*-Value
Age	1.12	1.009–1.2358	0.034
Hypertension	2.87	0.688–12.0003	0.148
Beta-lactam use	6.48	0.876–47.8604	0.067

## Data Availability

The data analysed in this study were obtained from the Tasmania Health Service and collected from patients’ records. Human research Ethics Committee and site authorisation approvals are required to access the data.
